# Comparison of the effect of saffron, crocin, and safranal on serum levels of oxidants and antioxidants in diabetic rats: A systematic review and meta‐analysis of animal studies

**DOI:** 10.1002/fsn3.3302

**Published:** 2023-03-13

**Authors:** Yaser Mohammadi, Azam Rezaei Farimani, Hossein Beydokhti, Seyed Mohammad Riahi

**Affiliations:** ^1^ Qaen School of Nursing and Midwifery Birjand University of Medical Sciences Birjand Iran; ^2^ Department of Biochemistry, Faculty of Medicine Birjand University of Medical Sciences Birjand Iran; ^3^ Department of General Courses, School of Medicine Birjand University of Medical Sciences Birjand Iran; ^4^ Department of Community Medicine, Cardiovascular Diseases Research Center Birjand University of Medical Sciences Birjand Iran

**Keywords:** crocin, diabetes, meta‐analysis, oxidative stress, saffron, safranal

## Abstract

This study aimed to evaluate the effect of saffron, crocin, and safranal on serum levels of oxidants and antioxidants in diabetic rats. The authors searched the databases with standard keywords until June 8, 2021. The random‐effects model was used to pool standardized mean differences (SMD) with 95% confidence intervals to assess the effects of saffron and its active component. To investigate heterogeneity, subgroup analysis and meta‐regression were utilized. Begg and Egger's tests were used to measure publication bias. Our results showed that saffron, crocin, and safranal were able to significantly reduce the serum levels of oxidants with strong efficacy so that saffron had the highest effectiveness on serum malondialdehyde (SMD, −2.84 (μmol/L) [95% confidence interval (CI), −4.32 to −1.36]; *p* < .001, *I* ^2^ = 83.5%). In addition, saffron and its effective compounds were highly effective by increasing the serum level of antioxidants. In addition, saffron and its effective compounds were able to significantly increase the serum level of antioxidants with strong efficacy, while saffron had the highest effect on the serum level of total antioxidant capacity (SMD, 3.90 (μmol/L) [95% CI, 0.78–7.03]; *p* = .014, *I* ^2^ = 86.9%). The findings of this study show that treatment with saffron, crocin, and safranal by strengthening the antioxidant defense system and modulating oxidative stress shows antidiabetic effects in the diabetic model of rats, also these findings support the potential effect of saffron and its effective compounds for the management of diabetes and its complications. However, more human studies are needed.

## Graphical abstract

To our knowledge, this is the first systematic review and meta‐analysis that has comprehensively investigated the effect of saffron and its active compounds on oxidative stress markers (oxidants and antioxidants). In this meta‐analysis, 15 studies were reviewed. The results of our study showed that saffron and its active compounds (crocin and safranal) with high effectiveness led to a reduction in oxidative stress caused by long‐term hyperglycemia. In addition, we showed that safranal is more effective than saffron and crocin.

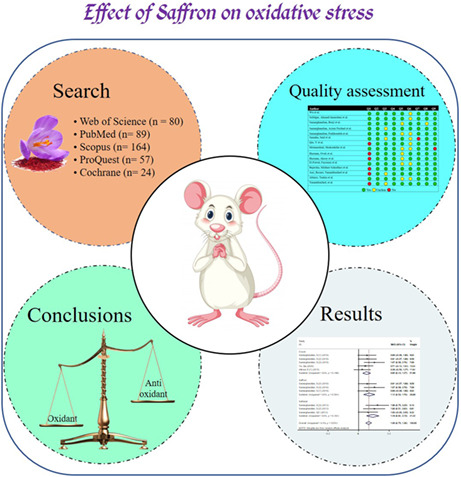

## 1 INTRODUCTION

Diabetes is a chronic, common, and multifactorial disease defined by hyperglycemia caused by reduced insulin production or insulin resistance, leaving the body unable to completely respond to insulin. By 2030, the number of individuals with diabetes is anticipated to reach 578 million (Saeedi et al., 2019). Type 1 diabetes mellitus and type 2 diabetes mellitus (T2DM) are the two most commonly recognized types of diabetes, with T2DM accounting for around 90% of all diabetes occurrences (Hussain et al., 2020). All cases of diabetes, if not adequately controlled, can develop diabetic complications, which are the leading cause of death and disability.

Although numerous studies have been conducted to clarify the molecular processes underlying the development of diabetic complications, the pathophysiology of these problems remains still unknown. Various studies have suggested that oxidative stress is important in the pathogenesis, progression, and consequences of diabetes (Rains & Jain, 2011; Yaribeygi et al., 2020). Oxidative stress is a prevalent occurrence in uncontrolled hyperglycemia. Prolonged uncontrolled hyperglycemia produces dangerous by‐products, such as reactive oxygen species (ROS), through a variety of pathways, including protein kinase C activation, glucose autoxidation, oxidative phosphorylation, and sorbitol formation (Rains & Jain, 2011). When the rate of free radical production exceeds the antioxidant defense mechanisms, oxidative stress occurs, resulting in free radical toxicity, in this situation, free radicals lead to apoptosis and dysfunction of various tissues including the kidney, liver, pancreas, brain, eyes, and irreversible damage including nephropathy, retinopathy, and neuropathy (Yaribeygi et al., 2020). Therefore, regulating the balance between oxidant and the antioxidant defense system is an important issue in diabetes. Conventional diabetes treatments are primarily done to regulate blood sugar levels and do not appear to have a positive effect on complications. In this regard, the use of natural remedies instead of chemical and pharmacological treatments is preferable.

Saffron (*Crocus sativus* L.) is a species of flowering plant of the *Crocus* genus in the iris family Iridaceae. Saffron is currently known to grow in the Mediterranean, East Asia, and Iran Region. Saffron is considered to be the most valuable spice by weight (Cardone et al., 2020). According to studies, saffron has anticancer, antidiabetic, antioxidant, memory and learning enhancing, and anti‐inflammatory effects (Xing et al., 2021). The pharmacological activities of saffron are attributed to its active constituents, including crocin, picrocrocin, and safranal. Evidence suggests that saffron might be used to treat diabetes (El Khoudri et al., 2021). As mentioned, there is a close relationship between hyperglycemia and oxidative stress. The results of studies have shown that saffron and its active compounds exert antioxidant effects by affecting various signaling pathways. Crocin significantly increases peripheral insulin sensitivity in peripheral insulin‐dependent cells (fat cells, muscle tissues, and heart) by phosphorylating acetyl cocarboxylase (AMPK/ACC) and mitogen‐activated protein kinases (Mohammadi et al., 2022). It also prevents diabetes‐induced apoptosis in pancreatic β‐cells by downregulating p53 protein, improves β‐cell function, and prevents blood sugar increase (Yaribeygi et al., 2018). In addition, saffron and its active compounds, by activating the Akt kinase signaling pathway, lead to GLUT‐4 being placed in the plasma membrane and increasing glucose absorption by insulin‐dependent tissues, resulting in a decrease in blood sugar (Mohammadi et al., 2023). As a result, saffron, crocin, and safranal exert antioxidant effects indirectly by reducing blood glucose. Although the hypoglycemic effects of saffron have been explored, the antioxidant benefits of saffron have not been well investigated. Therefore, the goal of this study was to see how saffron and its active components affected serum levels of oxidative stress markers in a diabetic rat model.

## 2 METHODS

The current systematic review and meta‐analysis investigated the impact of saffron and its active components on serum levels of oxidative stress markers in diabetes using the Cochrane Handbook for Systematic Reviews of Intervention criteria. The protocol of this study was registered in the Birjand University of Medical Sciences (BUMS). It has been approved before the start of the study with registration code *IR.BUMS.REC.1400.138*. The results were reported under the Preferred Reporting Items for Systematic Reviews and Meta‐Analyses (PRISMA) Guidelines (Moher, et al., 2015).

### 2.1 Strategy for searching

The details of database searches are shown in Table S1. Two independent researchers (Y.M. and H.B.) did a complete electronic search utilizing the five databases listed below through June 8, 2021, with no language restrictions (PubMed, Scopus, ProQuest, Web of Science, and Cochrane). Relevant keywords from Mesh and non‐Mesh phrases were used to search database sources. The search technique was based on Boolean search words (AND & OR). Furthermore, the reference lists of the selected papers (*n* = 1) were carefully verified. To ensure that the most recent articles in the field were obtained, a Google Scholar database search was performed.

### 2.2 Criteria for eligibility

Inclusion criteria: (1) animal studies, (2) investigations on the effects of saffron, crocin, and safranal on oxidative stress markers, and (3) studies on DM. Exclusion criteria: (1) randomized controlled trial studies, (2) studies on other diseases and other parameters, (3) conference abstracts, reviews, editorial or protocols, letters, case reports, and commentaries, (4) studies with incomplete data, (5) interventions by combining saffron and its constituents with other compounds or supplements.

### 2.3 Study selection and data extraction

Endnote software (version X9) was used to store all recognized papers from each database, with the Endnote function “Find duplicates” utilized to eliminate duplicates. After duplicate records were deleted, the remaining articles were assessed individually by two reviewers (Y.M. and A.R.F.) with a 97% agreement to validate their eligibility for inclusion. Any disputes were resolved by a face‐to‐face dialogue or consultation with a third reviewer throughout the study selection process (S.M.R.). There were two stages to the screening procedure. At first, the titles and abstracts were carefully reviewed and unrelated studies were excluded. In the second phase, the full‐text version of the remaining manuscripts was examined for eligibility. Finally, all eligible studies were included in this study. A specific form for data mining was created using Excel software. The following information was contained in the document: First author, year of publication, country of study, quality of the study, animal breed, sample size, duration of intervention, the dose of intervention (mg/kg/day), mean and standard deviation of finding before and after the intervention.

### 2.4 Quality assessment

Articles were evaluated using SYRCLE's Risk of Bias (RoB) tool. The RoB tool is specifically designed for animal studies (Hooijmans et al., 2014). The articles' data were categorized into three categories: high risk of bias (H), uncertain risk of bias (U), and low risk of bias (L). A “NO” judgment indicates a high risk of bias; the word “Unclear” was used when the text description was insufficient to assess the risk of bias; and a “Yes” judgment indicates a low risk of bias. Studies with quality scores of less than 60% were excluded due to a high risk of bias. Two reviewers (Y.M. and A.R.F.) independently conducted a quality assessment with 97% agreement. To resolve disagreements in scoring, the writers had discussions and contacted a third author (S.M.R.).

### 2.5 Statistical analysis

We assessed the metabolic effects of saffron, crocin, and safranal using standardized mean difference (SMD) with a 95% confidence interval (CI). A random‐effects model was used to estimate SMD. For interpreting the SMD, the following guidelines had been suggested by Cohen: small, SMD = 0.2; medium, SMD = 0.5; and large, SMD = 0.8 (Faraone, 2008). Heterogeneity was determined using *Q* and *I* ^2^ statistics. *I* ^2^ values more than 0.6 were considered heterogeneous. To deal with heterogeneity, subgroup analysis and meta‐regression techniques were applied (Mokhayeri et al., 2018; Riahi & Mokhayeri, 2017). The publication bias was determined using the Egger's regression test, which is a parametric method. A significant value was considered .1 in analyses with less than 10 studies. Stata software (Version 14.0) was used to analyze the data.

## 3 RESULTS

### 3.1 Search results

**FIGURE 1:**
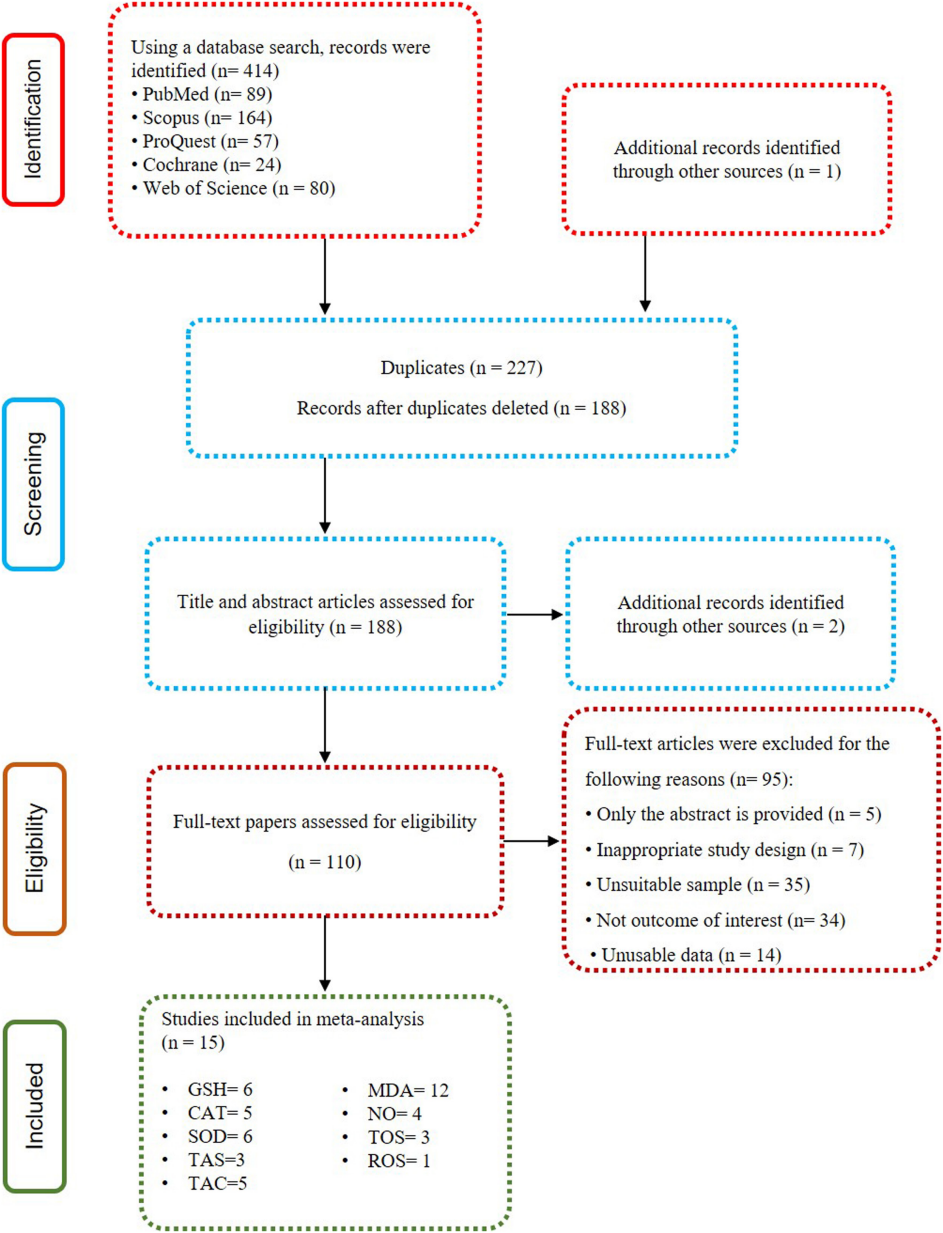
Flowchart of the study selection process for the systematic review and meta‐analysis.

The process of selecting articles is shown in detail in Figure 1. Overall, among 415 studies, 15 papers were evaluated for quality and were included in the current study.

### 3.2 Study characteristics

Table 1 shows the specs of 15 qualifying articles. These investigations were conducted between 2012 and 2020 in Iran, China, Egypt, Turkey, and Poland. There have been 14 studies on rats and one study on mice. To induce diabetes, 11 papers employed STZ (dose range 40–60 mg/kg), two papers used a high‐fat diet, and two papers used a combination of STZ and a high‐fat diet. The intervention for saffron, crocin, and safranal lasts between 14 and 70 days. Ten crocin articles (dose range 7.5–150 mg/kg), three saffron articles (dose range 10–100 mg/kg), and two safranal articles (dose range 0.25–0.75 mg/kg) were utilized.

**TABLE 1:** Characteristics of articles included in the meta‐analysis.

Reference	Year	Country	Animal	Sample size in each group	Type of diabetes induction/dosage (mg/kg)	Treatment period (day)	Type of intervention/dosage (mg/kg)	Measurement	Quality score
Wu et al. (2018)	2018	China	Rats	8	STZ/60	14	Crocin/60	MDA, SOD, NO	88.89
Sefidgar, Ahmadi‐hamedani et al. (2019)	2019	Iran	Rats	6	STZ/60	28	Crocin/60	TAS, TOS	88.89
Samarghandian, Borji et al. (2013)	2013	Iran	Rats	8	STZ/60	28	Safranal/0.25, 0.5, 0.75	MDA, GSH, SOD, CAT, NO	77.78
Samarghandian, Azimi‐Nezhad et al. (2016)	2016	Iran	Rats	9	STZ/60	28	Saffron/10, 20, 40	MDA, GSH, SOD, CAT, NO	88.89
Samarghandian, Azimi‐Nezhad, and Farkhondeh (2016)	2016	Iran	Rats	9	STZ/60	28	Crocin/10, 20, 30	MDA, GSH, SOD, CAT, NO	77.78
Samaha et al. (2019)	2019	Egypt	Rats	10	STZ/50	28	Crocin/10	MDA, GSH, SOD, TAC, CAT	66.67
Qiu et al. (2020)	2020	China	Mice	12	High‐fat diet	56	Crocin/50	MDA, GSH, SOD, CAT, ROS	77.78
Motamedrad et al. (2019)	2019	Iran	Rats	7	STZ/60	21	Saffron/25, 100	MDA, TAC	66.67
Hazman and Ovali (2015)	2014	Turkey	Rats	8	STZ (30)/High‐fat diet	28	Safranal	TAS, TOS	77.78
Hazman et al. (2016)	2016	Turkey	Rats	8	STZ (30)/High‐fat diet	42	Crocin/150	TAS, TOS	77.78
El‐Fawal et al. (2018)	2018	Egypt	Rats	8	High‐fat diet	70	Crocin/50	MDA	77.78
Bajerska et al. (2013)	2013	Poland	Rats	6	STZ/40	35	Saffron	MDA, TAC	88.89
Asri‐Rezaei et al. (2015)	2014	Iran	Rats	8	STZ/50	42	Crocin/12.5, 25, 50	MDA, TAC	77.78
Altinoz et al. (2015)	2015	Turkey	Rats	10	STZ/50	21	Crocin/20	MDA, GSH	77.78
Tamaddonfard et al. (2013)	2012	Iran	Rats	6	STZ/60	30	Crocin/7.5, 15, 30	MDA, TAC	77.78

Abbreviations: CAT, catalase; GSH, glutathione; MDA, malondialdehyde; NO, nitric oxide; ROS, reactive oxygen species; SOD, superoxide dismutase; TAC, total antioxidant capacity; TAS, total antioxidant status; TOS, total oxidant status.

### 3.3 Quality assessment

**FIGURE 2:**
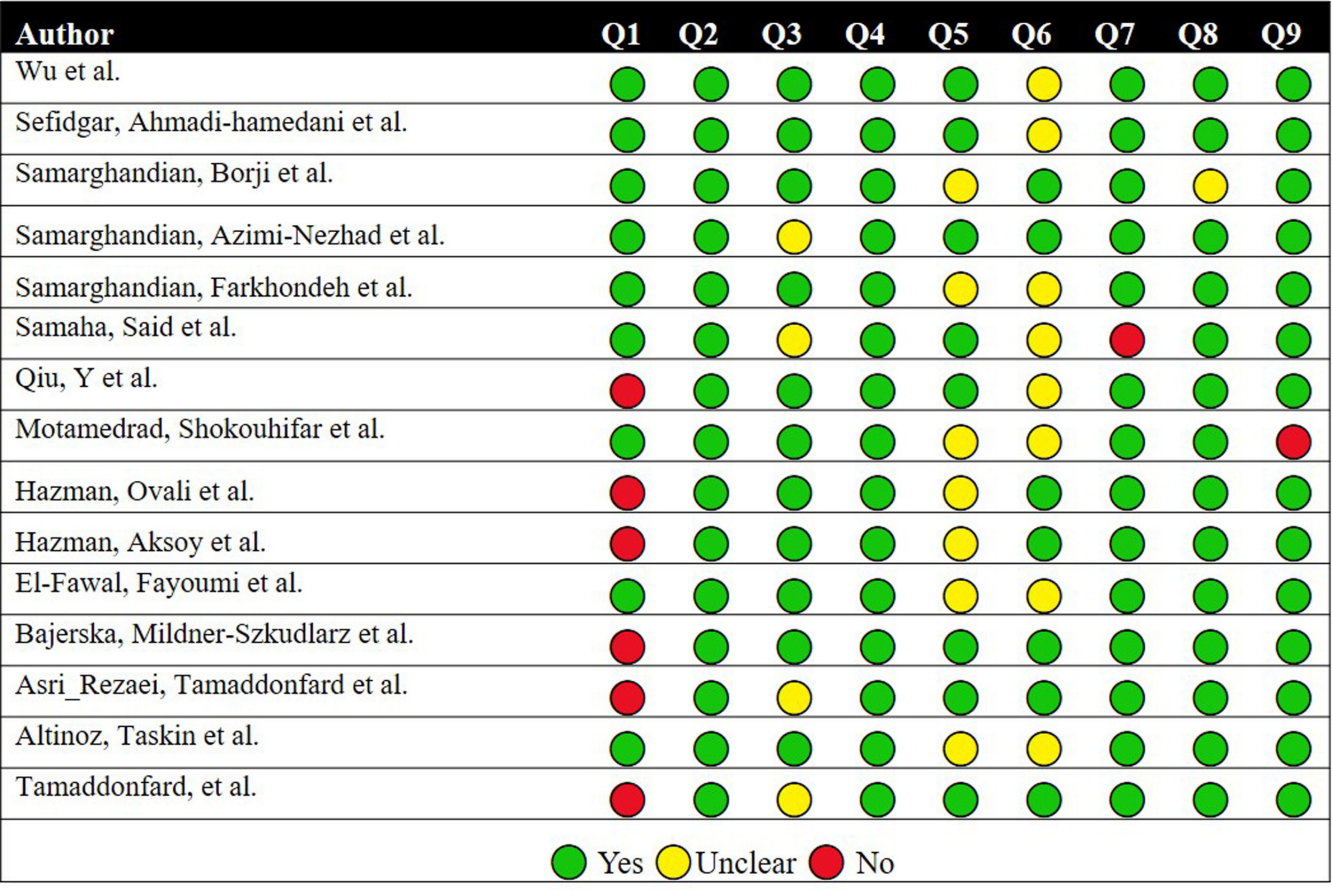
Quality assessment of included articles in the meta‐analysis. Q1: Was the allocation sequence adequately generated and applied? Q2: Were the groups similar at baseline, or were they adjusted for confounders in the analysis? Q3: Was the allocation to the different groups adequately concealed during? Q4: Were the animals randomly housed during the experiment? Q5: Were the caregivers and/or investigators blinded from knowledge of which intervention each animal received during the experiment? Q6: Was the outcome assessor‐blinded? Q7: Were incomplete outcome data adequately addressed? Q8: Are reports of the study free of selective outcome reporting? Q9: Was the study free of other problems that could result in a high risk of bias?

The results are shown in Table 2, and the specifics of the quality evaluation of the included articles are shown in Figure 2. Four studies had a score of 88.89, nine articles received a score of 77.78, and two papers received a score of 66.67 in the quality rating.

**TABLE 2:** Effect of saffron and its effective compounds on serum levels of oxidative stress markers.

Variable	*N*	SMD (95% CI)	*p*‐value for SMD	*Q*	*I* ^2^	Tau
Antioxidant markers						
GSH						
Saffron	3	1.12 (0.53, 1.70)	<0.001	1.37	0.0	0.00
Crocin	5	0.85 (0.43, 1.27)	<0.001	3.37	0.0	0.00
Safranal	3	1.49 (0.84, 2.15)	<0.001	1.53	0.0	0.00
CAT						
Saffron	3	0.75 (0.19, 1.30)	0.008	0.32	0.0	0.00
Crocin	4	0.77 (0.31, 1.24)	0.001	0.36	0.0	0.00
Safranal	3	1.49 (0.84, 2.15)	<0.001	0.93	0.0	0.00
SOD						
Saffron	3	1.29 (0.62, 1.97)	<0.001	2.52	20.7	0.07
Crocin	5	1.41 (0.58, 2.25)	0.001	12.27	67.4	0.59
Safranal	3	3.54 (2.59, 4.50)	<0.001	1.35	0.0	0.00
TAS						
Crocin	4	1.06 (−0.34, 2.47)	0.13	15.88	81.1	1.65
Safranal	2	0.43 (−2.32, 3.19)	0.75	12.06	91.7	3.62
TAC						
Saffron	3	3.90 (0.78, 7.03)	0.014	15.26	86.9	6.41
Crocin	7	1.82 (0.98, 2.67)	<0.001	14.50	58.6	0.74
Oxidant markers						
MDA						
Saffron	6	−2.84 (−4.32, −1.36)	<0.001	30.37	83.5	2.64
Crocin	13	−1.57 (−2.04, −1.11)	<0.001	23.42	48.8	0.34
Safranal	3	−1.90 (−3.86, 0.06)	0.057	14.04	85.8	2.54
NO						
Saffron	3	−1.58 (−2.72, −0.43)	0.007	6.44	68.9	0.70
Crocin	4	−1.65 (−2.52, −0.78)	<0.001	7.10	57.8	0.45
Safranal	2	−4.56 (−6.31, −2.82)	<0.001	1.52	34.4	0.56
TOS						
Crocin	4	−0.97 (−2.74, 0.79)	0.27	23.47	87.2	2.81
Safranal	2	−3.61 (−13.39, 6.18)	0.47	30.40	96.7	48.24
ROS						
Crocin	1	−0.80 (−1.63, 0.04)	0.06	0.00	0	0.00

Abbreviations: CAT, catalase; GSH, glutathione; MDA, malondialdehyde; NO, nitric oxide; ROS, reactive oxygen species; SMD, standardized mean difference; SOD, superoxide dismutase; TAC, total antioxidant capacity; TAS, total antioxidant status; TOS, total oxidant status.

### 3.4 Meta‐analysis results for the effect of saffron, crocin, and safranal on serum level of oxidative stress markers

Table 2 shows the results of the meta‐analysis. The diabetic group treated with saffron, crocin, and safranal was compared to the diabetic control group in this study.

#### 3.4.1 Antioxidant markers

**FIGURE 3:**
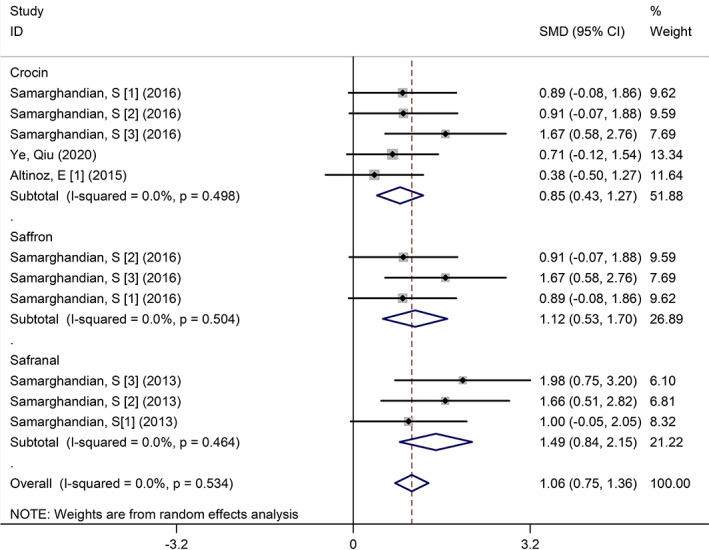
Subgroup analysis for glutathione compared with the control group. CI, confidence interval; SD, standard deviation; SMD, standardized mean difference.

The results of our meta‐analysis showed that in the treated group compared with the diabetic control group, the serum levels of antioxidants (glutathione [GSH], catalase [CAT], superoxide dismutase [SOD], and total antioxidant capacity [TAC]) increased significantly. The most effective effect of saffron on the serum levels of TAC (SMD, 3.90 (μmol/L) [95% CI, 0.78–7.03]; *p* = .014, *I* ^2^ = 86.9%) and crocin had the least effect on serum CAT levels (SMD, 0.77 [nmol/L] [95% CI, 0.31–1.24]; *p* = .001, *I* ^2^ = 0.0%). Figure 3 shows the subgroup analysis for GSH.

#### 3.4.2 Oxidant markers

According to our results, the serum levels of oxidants (malondialdehyde [MDA], nitric oxide [NO], and ROS) in the treated groups were significantly lower than those in the diabetic control group. However, in some analyses, substantial heterogeneity was observed. Our results show that saffron had the highest effectiveness on serum MDA (SMD, −2.84 (μmol/L) [95% CI, −4.32 to −1.36]; *p* < .001, *I* ^2^ = 83.5%) levels and crocin had the lowest effectiveness on ROS serum levels (SMD, −0.80 (U/mL) [95% CI, −1.63 to 0.04]; *p* = .06, *I* ^2^ = 0.0%).

#### 3.4.3 Meta‐regression

**FIGURE 4:**
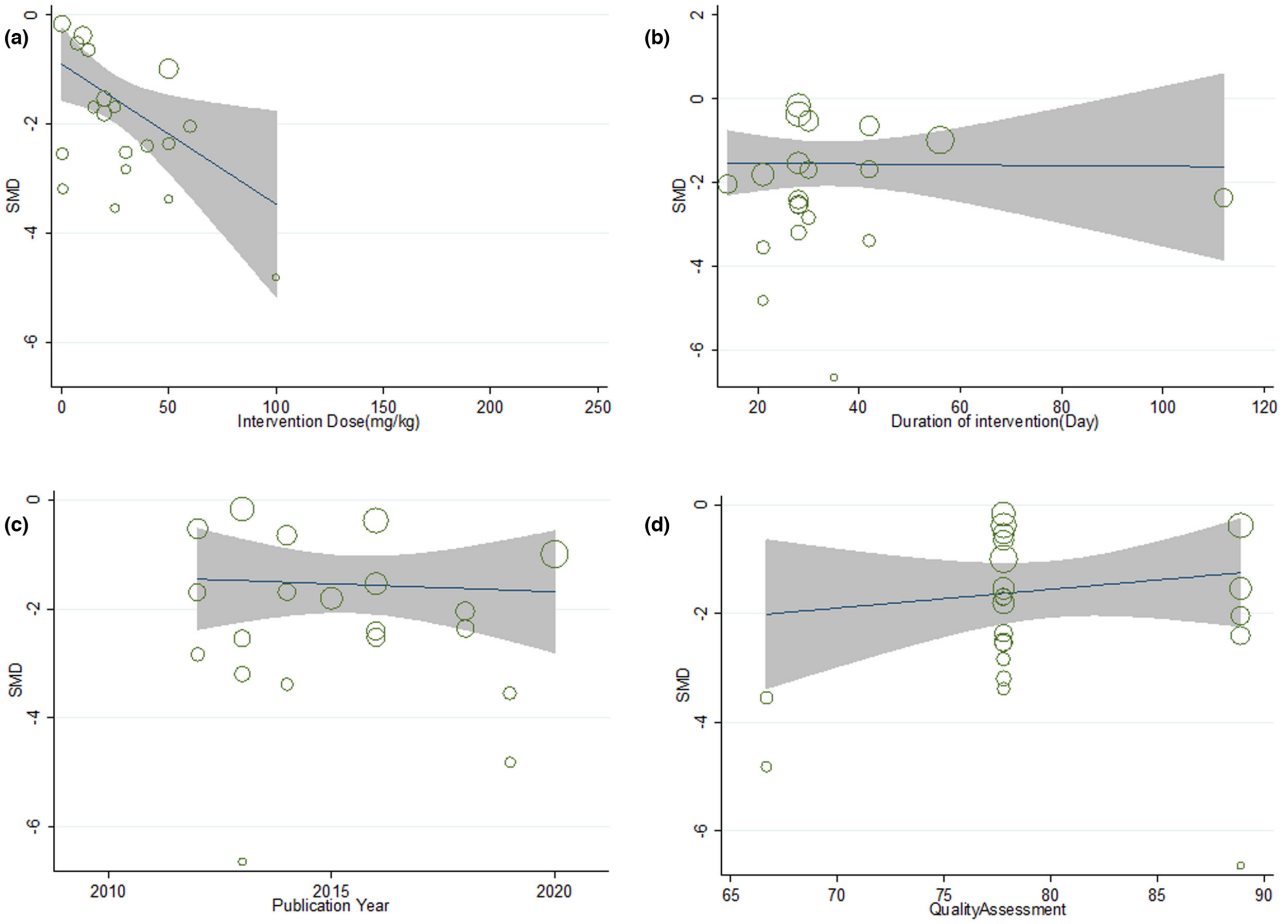
Meta‐regression for malondialdehyde based on (A) Intervention dose, (B) Duration of intervention, (C) Publication year, (D) Quality assessment.

In the present study, the dose and duration of intervention, publication year, quality score, and sample size meta‐regression were performed to evaluate the effect of quantitative covariates on each of the oxidative stress markers (oxidant and antioxidant). Figure 4 shows MDA meta‐regression. Meta‐regression for MDA showed that there is a significant relationship between the intervention dose (*b* = −0.027; [95% CI = −0.04 to −0.006]; *p* = .01) and the effectiveness of saffron and its active compounds, but there is no relationship between the duration of the intervention (*b* = 0.001; [95% CI = −0.030 to 0.032]; *p* = .94), publication year (*b* = −0.046; [95% CI = −0.30 to 0.21]; *p* = .7), and quality assessment (*b* = −0.011; [95% CI = −0.078 to 0.05]; *p* = .7). Other parameters of oxidative stress (oxidant and antioxidant) were analyzed, but no significant connection was found (data were not shown).

#### 3.4.4 Publication bias

**FIGURE 5:**
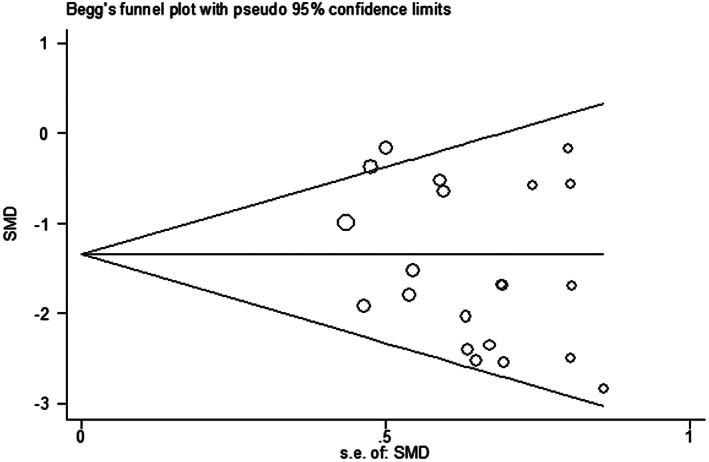
Funnel plot analysis for malondialdehyde index of all included studies. Begg's correlation test (*p* = .392) and Egger's test (*p* = .137).

In this study, publication bias was investigated using a funnel plot and an Egger's test, and no indication of publication bias was found (Figure 5).

## 4 DISCUSSION

### 4.1 Main findings

This is the first systematic review and meta‐analysis that we are aware of that looks at the effect of saffron and its active components on oxidative stress markers in the serum (oxidants and antioxidants).

In our study, the oxidant–antioxidant balance was examined by measuring the levels of markers oxidants and antioxidants in diabetic rats. According to the current study, an increase in oxidants (MDA, NO, total oxidant status, and ROS) and a reduction in antioxidants (SOD, GSH, CAT, total antioxidant status, and TAC) suggest a shift in the oxidant–antioxidant balance in diabetic rats. The results of our study showed that saffron, crocin, and safranal with strong efficacy significantly decreased the serum levels of oxidants and significantly increased the antioxidant, thus restoring the oxidant–antioxidant balance and regulating the state of oxidative stress.

Our results showed that saffron, crocin, and safranal were able to significantly reduce serum levels of MDA, NO, and ROS. Also, our results show a strong relationship between the effectiveness of saffron and its effective compounds and the reduction of oxidants. Although substantial heterogeneity was observed in some of the analyses, the 95% CI of the Pooled SMD was relatively narrow. In this study, the population studied in majority of studies was conducted on rats, and this issue could not be a source of heterogeneity. Factors affecting heterogeneity in this study include the small number of studies in some subgroup analyses as well as the intervention dose. Due to the small number of studies, it was not possible to examine the dose–response effect in this study, and it is suggested that the results of this study be updated in the coming years. Oxidative stress caused by an imbalance between oxidants and antioxidants plays an important role in the pathogenesis and complications of diabetes (Pisoschi et al., 2021). Hyperglycemia leads to the overproduction of oxygen‐free radicals that contribute to the progression of diabetes and its complications (Beloucif et al., 2021). Hyperglycemia appears to enhance mitochondrial glucose oxidation, resulting in a significant amount of superoxide and other free radicals being produced (Eftekharpour & Fernyhough, 2021). Lipid peroxides are formed when lipids react with ROS, and MDA is one of the products produced by lipid peroxides (Altinoz et al., 2015). MDA is an aldehydic product of lipid peroxidation that reacts rapidly with biological molecules such as lipids, proteins, and nucleic acids, leading to liver and β‐pancreatic cells dysfunction, thus impairing glucose regulation (Samarghandian et al., 2013). In recent years, MDA has become the most important and researched lipid peroxidation parameter, so MDA is used to evaluate the extent of lipid peroxidation (Altinoz et al., 2015). The results of our study showed that saffron and its active compounds significantly reduced the serum level of MDA, we also observed that saffron reduces MDA more effectively than crocin and safranal, although heterogeneity was high in the studies. Prolonged hyperglycemia activates the renin–angiotensin–aldosterone system, which accelerates NO metabolism, which impairs vasodilation, leading to endothelial damage and glomerular nephropathy (Abou‐Hany et al., 2018). Our results showed that saffron and crocin significantly reduced serum NO levels, indicating a protective effect of saffron and crocin in the management of diabetes complications.

Our results showed that saffron and its active compounds with high effectiveness were able to significantly increase serum levels of GSH, CAT, SOD, and TAC. The results also showed that safranal as the main agent of saffron aroma increases serum SOD levels more effectively than crocin and saffron, although the heterogeneity was slightly higher in some studies. To fight free radicals and limit their destruction, living organisms have developed antioxidant defense systems. Enzymes including GSH, CAT, and SOD are part of these antioxidant defense systems (Asri‐Rezaei et al., 2015). GSH reacts with free radicals and is an important substrate for glutathione‐S‐transferase and glutathione peroxidase, which are involved in cellular defense mechanisms (Samarghandian et al., 2017). SOD removes superoxide radicals (Hashemi et al., 2020), while CAT decomposes hydrogen peroxide into H_2_O and O_2_ (Margaritis et al., 2020); hence, these enzymes may play a role in plasma redox state regulation. Therefore, the results of our study show that saffron, crocin, and safranal reduce oxidative stress by strengthening the antioxidant defense system.

### 4.2 External validity

Our analysis shows that saffron and its active compounds with a potential effect in reducing oxidative stress are very reproducible in terms of the direction and extent of the effect in different species, which is useful for its translation to humans. However, there are some notable differences between animal models of diabetes and diabetic patients for whom treatment is intended, which we briefly mention. The most remarkable difference was comorbidities, in the studies included in the meta‐analysis, none of the animals suffered from comorbidities. This is while patients with diabetes may experience a wide range of comorbidities such as cardiovascular, renal, blood pressure, etc., which can aggravate oxidative stress. Also, animals are not age‐matched to diabetic subjects, so very few studies have been conducted on middle‐aged or elderly animals. With increasing age, the body's immune system weakens and reduces its ability against oxidative stress factors, so it may be susceptible to a wide range of diseases. Of the 15 studies included in our meta‐analysis, only one article used female animals, indicating a sex bias in these data. In addition, the animal studies that were examined in this meta‐analysis were different from each other in terms of the duration of the intervention period and the dosage used, which can of the most important external factors affecting the level of oxidative stress markers.

### 4.3 Strengths and limitations

The current study has some strengths: The first meta‐analysis study was about the effect of saffron and its components on serum levels of oxidants and antioxidants in animal studies. Also, we performed a subgroup analysis based on the type of intervention. In addition, there are a few limitations to our research that should be mentioned. First, the number of papers included in some analyses was minimal, particularly in ROS. Second, we observed high heterogeneity in some studies in our meta‐analysis. Studies included in a meta‐analysis of preclinical studies may not aim to measure a single treatment effect. However, the general purpose of animal studies is to evaluate differences in treatment effects under different conditions. So, they tend to be more heterogeneous than clinical trials. We attempted to identify sources of heterogeneity using meta‐regression and subgroup analysis techniques.

## 5 CONCLUSION

The findings of this study show that treatment with saffron, crocin, and safranal by strengthening the antioxidant defense system and modulating oxidative stress shows antidiabetic effects in the diabetic model of rats, also these findings support the potential effect of saffron and its effective compounds for the management of diabetes and its complications. However, more studies in human models are needed to accurately assess the protective mechanisms of saffron, crocin, and safranal against diabetes and its complications.

## FUNDING INFORMATION

This study was performed with the financial support of Birjand University of Medical Sciences (Grant No.: 5762).

## CONFLICT OF INTEREST STATEMENT

The authors declare that they have no conflict of interest.

## Supporting information


Table S1.

## Data Availability

We confirm that the data supporting the findings of this study are available within the article.
