# Supplementary material for: Comparison of the effect of saffron, crocin, and safranal on serum levels of oxidants and antioxidants in diabetic rats: A systematic review and meta‐analysis of animal studies
Source: Food Sci Nutr. 2023 Mar 13;11(6):2429–39. doi: 10.1002/fsn3.3302 (PMC10261797; doi:10.1002/fsn3.3302)
Supplement: Supplementary file 1 — Table S1. [file FSN3-11-2429-s001.docx]

**eTable 1.** Search strategy in databases

| **Databases** | **Search Strategy** | **Result** |
| --- | --- | --- |
| **Scopus** | ( TITLE-ABS-KEY ( saffron* ) OR TITLE-ABS-KEY ( safron* ) OR TITLE-ABS-KEY ( crocus* ) OR TITLE-ABS-KEY ( safrana* ) OR TITLE-ABS-KEY ( crocetin* ) OR TITLE-ABS-KEY ( picrocrocin* ) OR TITLE-ABS-KEY ( crocin* ) ) AND ( TITLE-ABS-KEY ( diabet* ) OR TITLE-ABS-KEY ( "Diabetic Nephropathies" ) ) AND ( TITLE-ABS-KEY ( antioxidan* ) OR TITLE-ABS-KEY ( oxidativ* ) OR TITLE-ABS-KEY ( "oxidative stress*" ) OR TITLE-ABS-KEY ( "Oxidative marker*" ) OR TITLE-ABS-KEY ( "Enzymatic oxidativ*" ) OR TITLE-ABS-KEY ( "anti-oxidative marker*" ) OR TITLE-ABS-KEY ( oxidant* ) OR TITLE-ABS-KEY ( "Free radical*" ) OR TITLE-ABS-KEY ( freeradical* ) OR TITLE-ABS-KEY ( malondialdehyd* ) OR TITLE-ABS-KEY ( "Superoxide dismutas*" ) OR TITLE-ABS-KEY ( glutathion* ) OR TITLE-ABS-KEY ( "Non-enzymatic oxidative*" ) OR TITLE-ABS-KEY ( "anti-oxidative marker*" ) OR TITLE-ABS-KEY ( catalas* ) OR TITLE-ABS-KEY ( "Selenoglutathione peroxidase*" ) ) | 164 |
| **PubMed** | ((Antioxidan*[Text Word] OR "oxidativ*"[Text Word] OR "oxidative stress*"[Text Word] OR “Oxidative marker*"[Text Word] OR “Enzymatic oxidativ*"[Text Word] OR “anti-oxidative marker*"[Text Word] OR Oxidant*[Text Word] OR "Free radical*"[Text Word] OR Freeradical*[Text Word] OR Malondialdehyd*[Text Word] OR "Superoxide dismutas* "[Text Word] OR Glutathion*[Text Word] OR “Non-enzymatic oxidative*"[Text Word] OR “anti-oxidative marker*"[Text Word] OR “Catalas*"[Text Word] OR “Selenoglutathione peroxidase*"[Text Word] OR "oxidative stress"[MeSH Terms] OR "Antioxidants"[Mesh] OR "Malondialdehyde"[Mesh] OR "Superoxide Dismutase"[Mesh] OR "Glutathione"[Mesh] OR “Catalase"[MeSH]) AND (Saffron*[Text Word] OR safron*[Text Word] OR "Crocus*"[Text Word] OR Safrana*[Text Word] OR crocetin*[Text Word] OR picrocrocin*[Text Word] OR crocin*[Text Word] OR "Crocus"[Mesh] OR "safranal" [Supplementary Concept] OR "picrocrocin" [Supplementary Concept] OR "crocin" [Supplementary Concept])) AND (Diabet*[Text Word] OR "Diabetic Nephropathies"[Mesh] OR "Diabetes Mellitus"[Mesh] OR "Diabetes Mellitus, Type 2"[Mesh]) | 89 |
| **Proquest** | ((ti(Saffron*) OR ab(Saffron*) OR su(Saffron*) OR ti(safron*) OR ab(safron*) OR su(safron*) OR ti(Crocus*) OR ab(Crocus*) OR su(Crocus*) OR ti(Safrana*) OR ab(Safrana*) OR su(Safrana*) OR ti(crocetin*) OR ab(crocetin*) OR su(crocetin*) OR ti(picrocrocin*) OR ab(picrocrocin*) OR su(picrocrocin*) OR ti(crocin*) OR ab(crocin*) OR su(crocin*)) AND PEER(yes)) AND ((ti(Diabet*) OR ab(Diabet*) OR su(Diabet*) OR ti("Diabetic Nephropathies") OR ab("Diabetic Nephropathies") OR su("Diabetic Nephropathies")) AND PEER(yes)) AND ((ti(Antioxidan*) OR ab(Antioxidan*) OR su(Antioxidan*) OR ti(oxidativ*) OR ab(oxidativ*) OR su(oxidativ*) OR ti(("oxidative stress")) OR ab(("oxidative stress")) OR su(("oxidative stress")) OR ti("Oxidative marker*") OR ab("Oxidative marker*") OR su("Oxidative marker*") OR ti("Enzymatic oxidativ*") OR ab("Enzymatic oxidativ*") OR su("Enzymatic oxidativ*") OR ti("anti-oxidative marker*") OR ab("anti-oxidative marker*") OR su("anti-oxidative marker*") OR ti(Oxidant*) OR ab(Oxidant*) OR su(Oxidant*) OR ti(("free radical" OR "free radicals")) OR ab(("free radical" OR "free radicals")) OR su(("free radical" OR "free radicals")) OR ti(Freeradical*) OR ab(Freeradical*) OR su(Freeradical*) OR ti(Malondialdehyd*) OR ab(Malondialdehyd*) OR su(Malondialdehyd*) OR ti(("superoxide dismutase")) OR ab(("superoxide dismutase")) OR su(("superoxide dismutase")) OR ti(Glutathion*) OR ab(Glutathion*) OR su(Glutathion*) OR ti("Non-enzymatic oxidative*") OR ab("Non-enzymatic oxidative*") OR su("Non-enzymatic oxidative*") OR ti("anti-oxidative marker*") OR ab("anti-oxidative marker*") OR su("anti-oxidative marker*") OR ti(Catalas*) OR ab(Catalas*) OR su(Catalas*) OR ti("Selenoglutathione peroxidase*") OR ab("Selenoglutathione peroxidase*") OR su("Selenoglutathione peroxidase*")) AND PEER(yes)) | 57 |
| **Web of Science** | TI=(Saffron*) OR AB=(Saffron*) OR AK=(Saffron*) OR TI=( safron*) OR AB=( safron*) OR AK=( safron*) OR TI=( Crocus*) OR AB=( Crocus*) OR AK=( Crocus*) OR TI=( Safrana*) OR AB=( Safrana*) OR AK=( Safrana*) OR TI=( crocetin*) OR AB=( crocetin*) OR AK=( crocetin*) OR TI=( picrocrocin*) OR AB=( picrocrocin*) OR AK=( picrocrocin*) OR TI=( crocin*) OR AB=( crocin*) OR AK=( crocin*) AND TI=( Diabet*) OR AB=( Diabet*) OR AK=( Diabet*) OR TI=("Diabetic Nephropathies") OR AB=("Diabetic Nephropathies") OR AK=("Diabetic Nephropathies") AND TI=( Antioxidan*) OR AB=( Antioxidan*) OR AK=( Antioxidan*) OR TI=( oxidativ*) OR AB=(oxidativ*) OR AK=( oxidativ*) OR TI=(“oxidative stress*”) OR AB=(“oxidative stress*”) OR AK=(“oxidative stress*”) OR TI=(“Oxidative marker*”) OR AB=(“Oxidative marker*”) OR AK=(“Oxidative marker*”) OR TI=(“Enzymatic oxidativ*") OR AB=(“Enzymatic oxidativ*") OR AK=(“Enzymatic oxidativ*") OR TI=(“anti-oxidative marker*") OR AB=(“anti-oxidative marker*") OR AK=(“anti-oxidative marker*") OR TI=( Oxidant*) OR AB=( Oxidant*) OR AK=( Oxidant*) OR TI=("Free radical*") OR AB=("Free radical*") OR AK=("Free radical*") OR TI=( Freeradical*) OR AB=( Freeradical*) OR AK=( Freeradical*) OR TI=( Malondialdehyd*) OR AB=( Malondialdehyd*) OR AK=( Malondialdehyd*) OR TI=("Superoxide dismutas*") OR AB=("Superoxide dismutas*") OR AK=("Superoxide dismutas*") OR TI=( Glutathion*) OR AB=( Glutathion*) OR AK=( Glutathion*) OR TI=(“Non-enzymatic oxidative*") OR AB=(“Non-enzymatic oxidative*") OR AK=(“Non-enzymatic oxidative*") OR TI=(“anti-oxidative marker*") OR AB=(“anti-oxidative marker*") OR AK=(“anti-oxidative marker*") OR TI=( Catalas*) OR AB=( Catalas*) OR AK=( Catalas*) OR TI=(“Selenoglutathione peroxidase*") OR AB=(“Selenoglutathione peroxidase*") OR AK=(“Selenoglutathione peroxidase*") | 80 |
| **Cochrane** | #1 (Saffron*):ti,ab,kw = 276  #2 (safron*):ti,ab,kw= 4  #3 (Crocus*):ti,ab,kw= 145  #4 (Safrana*):ti,ab,kw= 4  #5 (crocetin*):ti,ab,kw= 28  #6 (picrocrocin*):ti,ab,kw= 0  #7 (crocin*):ti,ab,kw= 75  #8 MeSH descriptor: [Crocus] explode all trees= 55  #9 #1 OR #2 OR #3 OR #4 OR #5 OR #6 OR #7 OR #8= 349  #10 (Diabet*):ti,ab,kw= 90985  #11 MeSH descriptor: [Diabetic Nephropathies] explode all trees= 1412  #12 MeSH descriptor: [Diabetes Mellitus] explode all trees= 30783  #13 MeSH descriptor: [Diabetes Mellitus, Type 2] explode all trees= 17348  #14 #10 OR #11 OR #12 OR #13= 91206  #15 (Antioxidan*):ti,ab,kw= 12679  #16 (oxidativ*):ti,ab,kw= 12436  #17 (oxidative stress*):ti,ab,kw= 10239  #18 (Oxidative marker*):ti,ab,kw= 3891  #19 (Enzymatic oxidativ*):ti,ab,kw= 214  #20 (anti-oxidative marker*):ti,ab,kw= 69  #21 (Oxidant*):ti,ab,kw= 2091  #22 (Free radical*):ti,ab,kw= 4533  #23 (Freeradical*):ti,ab,kw= 145  #24 (Malondialdehyd*):ti,ab,kw= 2998  #25 (Superoxide dismutas*):ti,ab,kw= 2198  #26 (Glutathion*):ti,ab,kw = 3559  #27 (Non-enzymatic oxidative*):ti,ab,kw= 52  #28 (anti-oxidative marker*):ti,ab,kw= 69  #29 (Catalas*):ti,ab,kw= 926  #30 (Selenoglutathione peroxidase*):ti,ab,kw= 1  #31 MeSH descriptor: [Oxidative Stress] explode all trees= 2978  #32 MeSH descriptor: [Antioxidants] explode all trees= 4812  #33 MeSH descriptor: [Malondialdehyde] explode all trees= 1252  #34 MeSH descriptor: [Superoxide Dismutase] explode all trees= 774  #35 MeSH descriptor: [Glutathione] explode all trees= 671  #36 MeSH descriptor: [Catalase] explode all trees=260  #37 #15 OR #16 OR #17 OR #18 OR #19 OR #20 OR #21 OR #22 OR #23 OR #24 OR #25 OR #26 OR #27 OR #28 OR #29 OR #30 OR #31 OR #32 OR #33 OR #34 OR #35 OR #36= 25778  #38 #9 AND #14 AND #37= 24 | 24 |
